# Black abalone (*Haliotis cracherodii*) population structure shifts through deep time: Management implications for southern California's northern Channel Islands

**DOI:** 10.1002/ece3.5075

**Published:** 2019-04-02

**Authors:** Hannah Haas, Todd J. Braje, Matthew S. Edwards, Jon M. Erlandson, Stephen G. Whitaker

**Affiliations:** ^1^ Rincon Consultants, Inc. Sacramento California; ^2^ Department of Anthropology California Academy of Sciences San Francisco California; ^3^ Department of Biology San Diego State University San Diego California; ^4^ Museum of Natural and Cultural History and Department of Anthropology University of Oregon Eugene Oregon; ^5^ Channel Islands National Park Ventura California

**Keywords:** applied archeology, historical ecology, shifting baselines

## Abstract

1. For over 10,000 years, black abalone (*Haliotis cracherodii*) were an important resource in southern California, first for coastal Native Americans, then beginning in the nineteenth century, as one of the state's first commercial shellfisheries. By 1993, after years of heavy fishing, rising sea surface temperatures (SST), and the spread of withering syndrome (WS), black abalone populations declined dramatically, resulting in the closure of the Alta California fishery. After nearly 25 years of management and recovery efforts, black abalone are showing signs of ecological rebound along some Channel Island shorelines. These include the presence of juvenile abalone and increasing densities, largely from data collected by Channel Islands National Park (CINP) monitoring efforts that began in 1985.
2. In an effort to apply deeper historical perspectives to modern fisheries management and restoration, we analyzed black abalone size data from San Miguel Island at prehistoric and historical archeological sites spanning the last 10,000 years and compared these populations to those described by CINP biologists between 1985 and 2013.
3. We found a statistically significant relationship between SST and black abalone size distributions during the ancient record, along with dramatic shifts in population size structure toward larger individuals between the nineteenth century and modern periods. A pattern of larger mean black abalone sizes was identified during warm SSTs, when compared against intervals of cooler SSTs.
4. Synthesis and applications. Our study provides a deep historical perspective of abalone population size distributions, patterns within these distributions through time, and parallels to modern abalone populations. Our results may help managers determine whether the current (and future) size and age structure of intertidal black abalone populations around the northern Channel Islands are “natural” and healthy, measured against the 10,000 year history of black abalone fishing in southern California.

## 1 INTRODUCTION

The long‐term impacts, both positive and negative, of humans on local environments have become an important research avenue for a variety of disciplines over the last decade (e.g., Balée, 2006; Erlandson & Rick, 2010; Rick & Lockwood, 2012). Seminal research by fisheries biologists has shown that nearshore marine ecosystems are highly susceptible to anthropogenic impacts and effective modern management requires deep historical perspectives of human–marine ecodynamics (e.g., Pauly, 1995; Jackson et al., 2001). Archeologists and other historical scientists have taken up this call as part of a marine historical ecology research agenda, simply defined as the integration of marine ecology and history (Rick & Lockwood, 2012). One especially fruitful avenue of marine historical ecology studies has focused on the effects of humans on nearshore shellfish communities, incorporating archeological, paleoecological, and historical data into modern management practices and restoration strategies (e.g., Braje, Rick, Erlandson, Rogers‐Bennett, & Catton, 2016; Finney, Gregory‐Eaves, Douglas, & Smol, 2002; Lotze et al., 2006; Rick et al., 2016; Rogers‐Bennett, Haaker, Huff, & Dayton, 2002).

Along the Pacific Coast of North America, black abalone (*Haliotis cracherodii*) is an interesting species for historical ecology studies. California black abalone are critically endangered, and their management might benefit from deep historical perspectives. Since 1993, recreational and commercial black abalone fishing has been suspended, and despite careful management and restoration attempts, black abalone were upgraded by the National Marine Fisheries Service from a species of concern to endangered in 2009 under the Endangered Species Act (California Fish & Game Commission, 2005; Neuman, 2009). On San Nicolas and Santa Cruz islands, there have been some signs of recovery (e.g., recruitment and population expansion; Butler et al., 2009), and recruitment has been observed for black abalone at Anacapa, Santa Rosa, and San Miguel islands in recent years (Raimondi, Jurgens, & Tinker, 2015; S. G. Whitaker, personal obs.), but we lack a clear picture of what a “healthy” abalone population looked like prior to population collapse. Modern data only reflect black abalone sizes and densities following decades of intensive commercial harvest, widespread disease, and the local extirpation of southern sea otters (*Enhydra lutris*), one of the primary predators of black abalone. Some ecologists, however, have begun to incorporate historical and archeological datasets in the development of deep historical baselines to evaluate modern abalone populations (e.g., Rogers‐Bennett et al., 2002).

Here, we extend historical ecological studies of Channel Islands black abalone populations by compiling the size structure of black abalone collected from the rocky intertidal zone around San Miguel Island over the last 10,000 years. We use the maximum shell length of black abalone shells from archeological sites spanning the Early (11,700–7,500 cal BP), Middle (7,500–3,500 cal BP), and Late (3,500 cal BP–AD 1782) Holocene, and the historic period (ca. AD 1850–1915) to examine how population size structure changed through deep time and evaluate whether these changes can be attributed to anthropogenic and/or environmental causes. We then compare archeological and historical data to modern size data collected by Channel Islands National Park (CINP) biologists to assess whether the modern (and future) size and age structures of intertidal black abalone populations are similar compared against 10,000 years of continually and intensively harvested abalone populations. In doing so, our data may aid resource managers and restoration biologists in evaluating the health of current and future black abalone populations in southern California.

### 1.1 Environmental setting and cultural background

San Miguel Island is the western‐most and second smallest (37 km^2^) of the Northern Channel Islands (NCI). Approximately half of San Miguel's 43 linear km of coastline consists of rocky shores that support abalone and other shellfish species, although the length and extent of rocky shore habitats have changed with fluctuations in sea level and dune building (Erlandson, Rick, & Peterson, 2005; Graham, Dayton, & Erlandson, 2003).

Long‐term changes in sea surface temperature (SST), patterns of upwelling, marine productivity, and kelp forest cover (Graham et al., 2003; Kennett, 2005) are natural changes that contribute to variation in shellfish productivity and growth rates. Studies of varved sediments from the Santa Barbara Basin provide a high‐resolution record of Holocene SST changes and a picture of long‐term climatic variability, with resolution at 25‐year intervals for the last 3,000 years and 50‐year intervals for the rest of the Holocene (Kennett, 2005). During the Holocene, four cold‐water intervals (spanning 9,600–8,200 cal BP; 6,300–5,900 cal BP; 3,800–2,900 cal BP; and 1,500–500 cal BP) and four warm‐water intervals (spanning 11,000–9,600 cal BP; 8,200–6,300 cal BP, 5,900–3,800 cal BP; and 2,900–1,500 cal BP) occurred, and since ~500 years ago, SSTs have undergone general warming. Short‐term changes, such as El Niño‐Southern Oscillation (ENSO) events, are difficult to gauge, but are assumed to have occurred in the past, although probably at lower frequencies than today (Kennett, 2005).

The NCI and the coastal mainland were continuously occupied by the Chumash and their ancestors for at least 13,000 years. From the terminal Pleistocene through the Holocene, Native Channel Islanders were maritime hunter‐gatherers, who relied on marine foods, particularly nearshore shellfish, as their primary protein source (Braje, Rick, & Erlandson, 2012; Erlandson, Rick, & Braje, 2009). The resulting disposal of shellfish remains in large, coastal shell middens provide researchers with an excellent record of past shellfish harvesting activities and the structure and nature of shellfish populations through time.

While the archeological record does reflect human choice, it can provide a window into the ancient structure of local intertidal ecosystems. Prehistoric hunter–gatherers were driven by subsistence goals in their harvest pursuits and archeologists operationalize what resources they would have pursued using optimal foraging theory (OFT) and human behavioral ecology (HBE). OFT is a useful tool for considering the productivity of different prey choices through time (see Braje, Kennett, Erlandson, & Culleton, 2007). Studies using OFT and ethnohistoric records suggest that black abalone were a highly ranked resource throughout the Holocene based on their large size, meat yield, ease of capture, abundance, and aggregation. When available in the local intertidal system, hunter–gatherers would have targeted black abalone before nearly any other shellfish species. Although island shell middens often contain a range of black abalone shell sizes, Channel Islanders likely focused on larger individuals because they provide higher meat yields and are often less difficult to access than smaller abalone that often utilize crevice habitat. Thus, we assume that the archeological record of black abalone size will be biased toward adult abalone with juveniles underrepresented and the exclusion of post‐larvae abalone. This should be similar for modern monitoring data as post‐larvae, and juveniles are as difficult to access today as they would have been in the past.

Terminal Pleistocene (ca. >11,700 cal BP) and Early Holocene archeological sites are relatively uncommon when compared to Middle and Late Holocene sites. Many terminal Pleistocene/Early Holocene sites probably have been lost due to rising sea levels and other taphonomic processes, and most of the early sites on San Miguel consist of low density shell middens and lithic scatters (Rick, Erlandson, Vellanoweth, & Braje, 2005). Zooarchaeological analyses indicate that Early Holocene subsistence economies focused on rocky intertidal shellfish species such as California mussel and black abalone (Rick et al., 2005). By the Middle Holocene, the number of recorded NCI sites increases and evidence for large populations settled in permanent shoreline villages is apparent (Kennett, 2005). During the Late Holocene, there were significant alterations to subsistence, settlement patterns, and technology and a marked increase in populations (Erlandson et al., 2009). Many Late Holocene sites are large, permanently occupied coastal villages and high population densities led to the increased dependence on finfish (Kennett, 2005; Rick, Vellanoweth, Erlandson, & Kennett, 2002). Although the relative contribution of shellfish to the protein diets of the Late Holocene Chumash decreased, harvesting of shellfish intensified and the mean size of several key dietary species declined significantly (Braje et al., 2012).

After the last of the Island Chumash were removed to Spanish settlements on the mainland by AD 1822, Chinese fishermen established commercial fishing camps in Alta and Baja California in the 1850s focused primarily on the harvesting of intertidal black abalone (Braje, 2016). Chinese fishermen traveled from mainland bases to the Channel Islands to gather, dry, and process abalone for export, facilitated by spectacular abalone abundance resulting from local sea otter extirpation and the removal of Chumash harvesting pressure. Between the mid‐1850s and the late AD 1880s, Chinese fishermen harvested hundreds of tons of abalone from coastal waters each year (Braje, 2016). The fishery peaked in 1879 when an estimated 1,860 tons of abalone were harvested from southern Californian waters (Rogers‐Bennett et al., 2002). By the late nineteenth century, the Chinese fishery was in decline due to growing anti‐Chinese sentiment and passage of targeted, racist laws and, perhaps, declining intertidal abalone populations (Braje, 2016). Japanese immigrants filled the void and shifted the focus to subtidal abalone species by ~1898, taking as many as 40–50 dozen red (*Haliotis rufescens*), green (*H. fulgens*), and pink (*H. corrugata*) abalone per day. Black abalone were considered undesirable compared to other species and fished primarily as bait for most of the 20th century (Altstatt et al., 1996; Parker, Kaaker, & Togstad, 1992), until declines in subtidal species stocks led to the redevelopment of a commercial export fishery in 1968. The black abalone fishery experienced a rapid rise and peaked in 1973, followed by a severe harvest decline due to heavy fishing pressure, coastal development, and pollution (Altstatt et al., 1996). The appearance of withering foot syndrome (WS) in the late 1980s exacerbated declines in black abalone populations (Altstatt et al., 1996; Raimondi, Wilson, Ambrose, Engle, & Minchinton, 2002; Vilchis et al., 2005), and, in the mid‐1990s, California began enacting fishing closures to preserve remnant populations. All commercial and recreational black abalone fishing was closed in 1993 (Rogers‐Bennett et al., 2002), and despite extensive restoration efforts, black abalone were added to the U.S. Endangered Species list in 2009 (California Fish & Game Commission, 2005; Neuman, 2009).

### 1.2 Abalone ecology

Black abalone are a smooth‐shelled species of abalone that live in the intertidal and shallow subtidal zones of rocky reefs, rarely venturing into deeper waters (Tissot, 1992). Black abalone, like other abalone species, are broadcast spawners and commonly found aggregating (Ault, 1985). Growth rates for *Haliotis* spp. are rapid during early years, but slow as age and size increase (Parker et al., 1992). Black abalone, in particular, are considered juveniles until they reach 45–50 mm in length and, on average, grow to around 115 mm, with some reaching a maximum of 215 mm (Ault, 1985).

Environmental conditions seriously affect the health of abalone populations, directly and indirectly. Inhabiting an environment in which land and sea overlap, black abalone are susceptible to environmental changes affecting both the ocean and atmosphere (Harley & Rogers‐Bennett, 2004). California abalone species are linked to kelp forest ecosystems as giant kelp (*Macrocystis pyrifera*) is one of their primary food sources and, when coupled with red algae, is thought to produce the fastest growth rates (Leighton & Boolootian, 1963). Warmer water temperatures are typically correlated with fewer nutrients in the water column (Edwards & Estes, 2006), resulting in lower qualities and densities of kelp and, thus, a reduced food source (Vilchis et al., 2005). Consequently, warm SSTs are linked to reduced abalone growth rates and reproductive success, and an increase in the expression of WS (Harley & Rogers‐Bennett, 2004).

Several factors limit California abalone populations. Their slow growth rate and preferred habitat in the intertidal and shallow subtidal make them susceptible to terrestrial and marine predators, including ocher sea stars, octopi, cabezon, crabs, spiny lobsters, and particularly humans and sea otters (Harley & Rogers‐Bennett, 2004). The historical extirpation of otters from Channel Islands waters allowed abalone populations to explode, reaching densities of up to 100 individuals per square meter.

A catastrophic decline of black abalone in southern California occurred in the late 1980s through the mid‐1990s as WS, a Rickettsiales‐like disease, systematically eradicated entire populations (Altstatt et al., 1996; Raimondi et al., 2002). Commercial fishermen first recognized signs of the disease, empty shells, and deteriorated black abalone along the south shore of Santa Cruz Island in 1985. A year later, diseased abalone were observed and documented by CINP biologists on Anacapa Island. Subsequently, the disease was recognized throughout the Channel Islands and the southern California mainland (Altstatt et al., 1996; Harley & Rogers‐Bennett, 2004; Raimondi et al., 2002). Some studies indicate that the effects of WS are intensified by stressful environmental conditions such as warm SST (Davis, Richards, Haaker, & Parker, 1992; Tissot, 1988, 1990) and reduced food supply (Tegner & Dayton, 1987), although Lafferty and Kuris (1993) found no association between die‐offs and warm SST or changes in kelp density. The disease does not target any particular age or size class, and in areas where mass die‐offs have occurred, very few individuals remain (Raimondi et al., 2002).

Declines in abalone populations are assumed to have further led to a lack of success in juvenile recruitment likely due to low densities of reproductive adults which, in turn, results in reduced larval dispersal and the loss of suitable habitat, caused by the colonization of encrusting species (Raimondi et al., 2002). Because current recruitment patterns do not appear to be sufficient to replenish mainland populations of black abalone in southern California and WS continues to impact the existing population, several management and restoration strategies have been proposed (Moore, Finley, Friedman, & Robbins, 2002; Raimondi et al., 2002; Rogers‐Bennett et al., 2002; Vilchis et al., 2005). Restoration activities have included the establishment of protected areas, the relocation of existing populations to create high density areas of black abalone that may result in increased reproduction and recruitment, and the outplanting of larvae, juveniles, or adults reared in hatcheries (Moore et al., 2002).

## 2 MATERIALS AND METHODS

**Figure 1:**
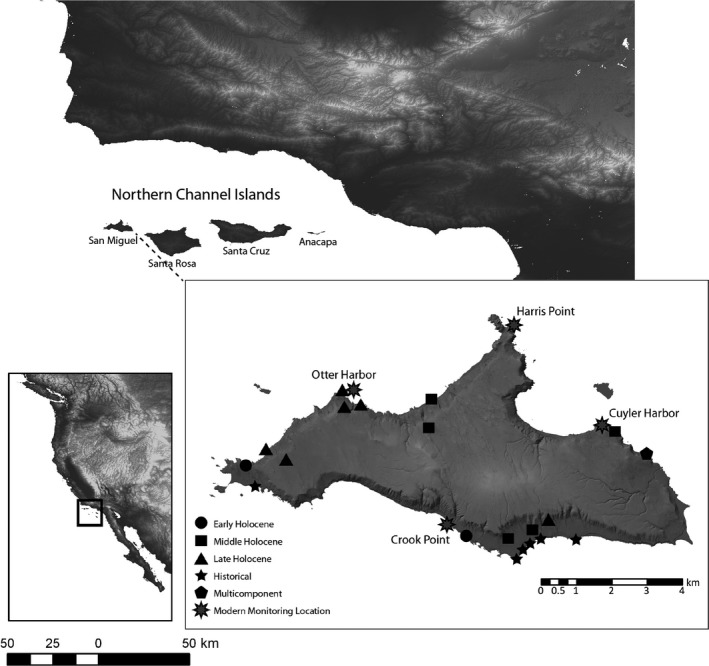
Location of the Santa Barbara Channel region, southern California, and the Northern Channel Islands. Inset map of San Miguel Islands and the locations of archeological sites and modern monitoring locations used in this study

We analyzed San Miguel Island black abalone shell measurements from archeological sites spanning the last 10,000 years (Erlandson, Rick, Braje, Steinberg, & Vellanoweth, 2008), and modern black abalone shell measurements collected by CINP biologists using fixed plots and a timed‐search protocol at monitoring locations between 1985 and 2013 (Table 1; Figure 1). We compiled 1,986 archeological black abalone shell measurements from 26 ancient or historical archeological sites spanning 10,000 years. Additional measurements were available, but excluded based on their small sample size (*n* < 15). We included only measurements from whole or nearly whole shells and for which total length could be accurately estimated. Shells were measured in the field and in the lab, from a combination of excavated assemblages and shells eroding from archeological sites. For field localities, care was taken to only measure surface shells that could be confidently correlated to a dated site component and to measure all the available black abalone shells. Archeological sites span the Early, Middle and Late Holocene, and the Historic Period, and the age of each prehistoric component was determined via radiocarbon (^14^C) dating of single marine shells or charred twigs, with dates calibrated to calendar years using the CALIB 5.0.2 program (Stuiver, Reimer, & Reimer, 2005). Historic Period sites were established based on correlations to known site types, ^14^C dating, or association with diagnostic artifacts.

**Table 1:** Sample size and black abalone length measurements for temporal periods and sea surface temperature intervals^a^

Period	Age	*n*	Mean length (mm)	*SD*	Median length (mm)	Min length (mm)	Max. length (mm)
Temporal periods							
Early	10,000–7,500 BP	242	76.791	20.994	73	18	135
Middle	7,500–3,500 BP	535	96.841	28.075	97	19	165
Late	3,500–150 BP	594	74.117	21.566	72	15.4	151
Historical	150 BP	512	127.75	19.315	130	31	176
Modern	AD 1985–2013	20,495	97.247	29.945	101	10	188
Sea surface temperature intervals							
Warm	11,000–9,600 BP	67	81.731	22.362	80	29	135
Cold	9,600−8200 BP	175	74.899	20.193	72	18	127
Warm	8,200–6,300 BP	61	84.426	19.128	82	44	131
Cold	6,300–5,900 BP	25	60.52	19.498	61	19	107
Warm	5,900–3,800 BP	449	100.55	27.6	101	33	165
Warm	2,900–1,500 BP	240	82.683	20.458	82	32	151
Cold	1,500–500 BP	330	67.769	20.058	64.5	15.4	131
Warm	500−150 BP	537	125.426	22.255	130	31	176
Warm	AD 1985–2013	20,495	97.247	29.944	101	10	188

a SST interval information from Kennett and Kennett (2000).

Black abalone size data were compared among time periods according to Early Holocene (*n* = 242), Middle Holocene (*n* = 535), Late Holocene (*n* = 594), Historical (*n* = 513), and Modern (*n* = 20,495) temporal divisions. Data also were compared among SST periods, according to warm intervals (11,000–9,600 BP, *n* = 67; 8,200–6,300 BP, *n* = 61; 5,900–3,800 BP, *n* = 449; 2,900–1,500 BP, *n* = 240; and general warming since 500 BP, archeological *n* = 537; modern *n* = 20,495) and cold intervals (9,600–8,200 BP, *n* = 175; 6,300–5,900 BP, *n* = 25; 3,800–2,900 BP, no data; and 1,500–500 BP, *n* = 330). Archeological and modern data for the last warm interval (500 BP to present) were treated separately to compare modern data to data from the last 10,000 years.

To compare mean shell sizes across time and SST periods, we conducted tests for homogeneity of variances using the Levene tests, and separate one‐way analyses of variance (ANOVAs), followed by Games–Howell post hoc tests to determine if the differences between each temporal period and/or each SST interval were statistically significant. To estimate the amount of variation in abalone shell sizes that were due to differences among the three temporal periods of the Holocene (Early, Middle, Late) versus simple natural variation within each temporal period, we estimated the magnitude of effects (*ω* ^2^) associated with each factor according to Graham and Edwards (2001). Here, we considered temporal period a fixed factor and used the average sample size for the three periods in the calculations. We then estimated how the importance of temporal period changed by repeating this procedure while including shell sizes from the Historic Period. To compare population distributions through time, we conducted Kolmogorov–Smirnov (KS) tests for each pair of periods to assess differences in population distribution across temporal and SST periods (Zar, 2014). Data were plotted on histograms to visually compare distributions when significant differences were identified. Data also were evaluated on the basis of juveniles versus adults. We considered all abalone shells <50 mm long to be juveniles and those >50 mm to be adults (Miner, Altstatt, Raimondi, & Minchinton, 2006). Both the ANOVAs and KS tests were conducted on data with juveniles removed to determine if the contribution of juveniles affected the outcome of the results; the removal of juveniles did not change the outcome of the tests. However, we assessed the contribution of juveniles over time using a contingency table, and conducted a Pearson's chi‐square analysis to assess whether age group was dependent on temporal period or SST interval. Percentages were then graphed; all statistical analyses were conducted using SPSS and SYSTAT (ver. 12) software.

## 3 RESULTS

**Figure 2:**
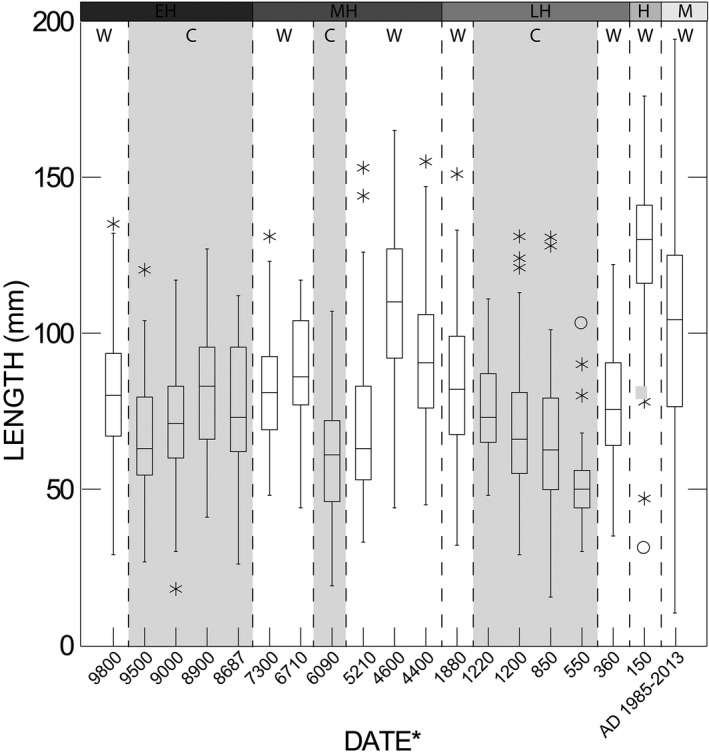
Boxplot showing all black abalone measurement data (mm) for each temporal period and sea surface temperature interval. *Note*: C: cold‐water interval; EH: Early Holocene; H: Historic Period; LH: Late Holocene; M: Modern; MH: Middle Holocene; W: warm‐water interval. (*Date is approximate midpoint in cal BP unless otherwise noted)

**Figure 3:**
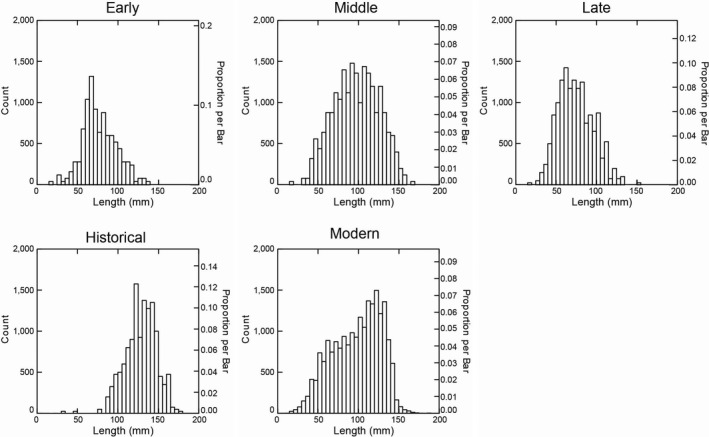
Histograms depicting black abalone population size structure during each temporal period. *Note*: Systat automatically bootstraps histogram data so that the scales are the same for each and can be compared visually

Unlike several other high‐ranked shellfish species, black abalone sizes (mean length) remained relatively stable throughout the Native American fishery of the Holocene (Erlandson et al., 2008; see Table 1, Figure 2). Indeed, differences among the Early, Middle, and Late Holocene periods accounted for only 15% of the observed variance in abalone sizes while natural variation within each temporal period accounted for 85% of the variance. This pattern changed during the Historic Period, when abalone were released from their main predators, humans, and sea otters, for three decades and shell sizes sharply increased. Consequently, when the Historic Period was included in the analysis, differences among the four periods (Early, Middle and Late Holocene, and the Historic Period) accounted for 53% of the observed variance in abalone sizes while natural variation within each period accounted for 47% of the variance in abalone sizes. Further, although the Games–Howell test revealed statistically significant differences between all pairs of periods (post hoc tests: *p* < 0.01 for all pairwise comparisons; Supporting Information Figure S1, Table S1), visual inspection of the data suggested no clear trends were evident among periods (Supporting Information Figure S1). We believe this was due to our large sample sizes (100s) and the ability to statistically resolve very small differences in shell sizes. We also observed statistically significant differences in both the variances (Leven test, *F*‐ratio = 34.909, *p* < 0.001) and size distributions (KS tests: *p* < 0.01 for each comparison) of black abalone among the four periods that were also evident with visual inspection of the data (Supporting Information Table S2; Figure 3). Specifically, the distribution of abalone sizes during the Early Holocene exhibited low variability (*σ* = 20.994) and skewed toward smaller abalone, with highest frequencies occurring between 50 and 100 mm. Abalone sizes were more variable during the Middle Holocene (*σ* = 28.075) and approximated a normal distribution, with abalone sizes peaking at approximately 100 mm. Abalone sizes during the Late Holocene resembled those of the Early Holocene in both variability (*σ* = 21.566) and distribution, skewing toward smaller sizes, with the highest frequencies again occurring between 50 and 100 mm. Following this, abalone sizes during the Historic Period show a substantial departure from those of the Late Holocene in both variability and distributions. Specifically, they were the least variable (*σ* = 19.315) and skewed toward larger abalone sizes, peaking between ~125 and 150 mm. Lastly, when we examined abalone during the Modern Period, we found them to be the most variable (*σ* = 29.945) and appeared more uniform in distribution, although they did tend to skew slightly toward larger sizes, peaking between 100 and 150 mm (Figure 3). For a summary of data organized by archeological site and/or locus, see Erlandson et al. (2008).

**Figure 4:**
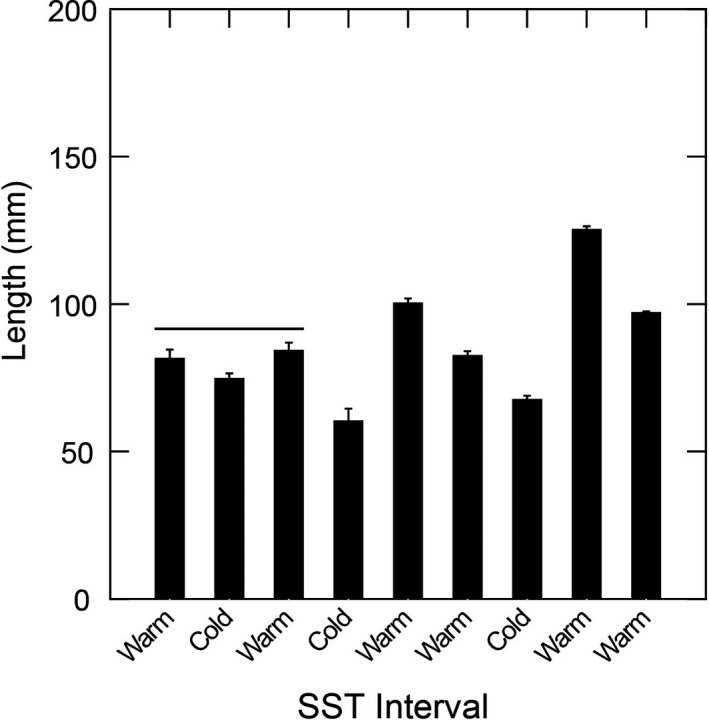
Mean black abalone size (length, mm) through time by SST interval. The *x*‐axis is in chronological order from oldest (far left) to most recent (far right). Pairs of means grouped by a horizontal line are not significantly different. Error bars indicate standard error. SST: sea surface temperature

**Figure 5:**
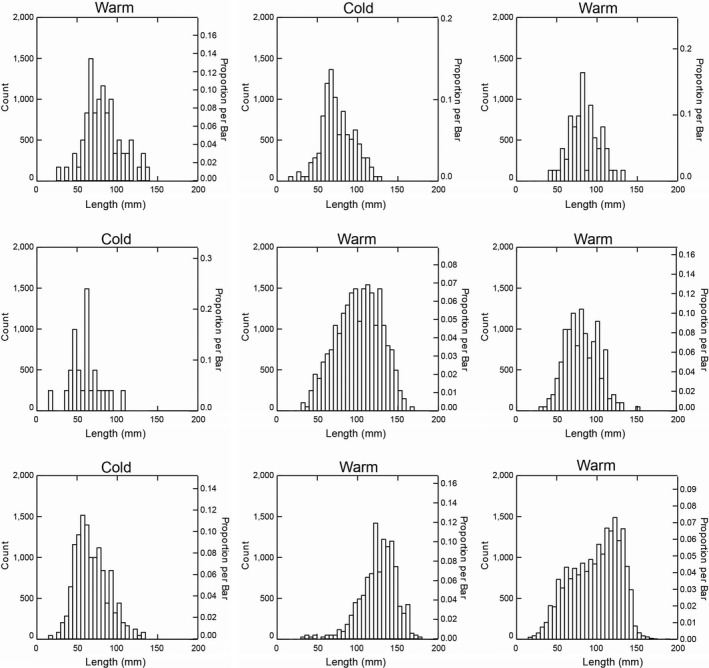
Histogram depicting black abalone population size (length, mm) structure across sea surface temperature intervals. Histograms are in chronological order from oldest (top left) to most recent (bottom right). *Note*: Systat automatically bootstraps histogram data so that the scales are the same for each and can be compared visually

**Figure 6:**
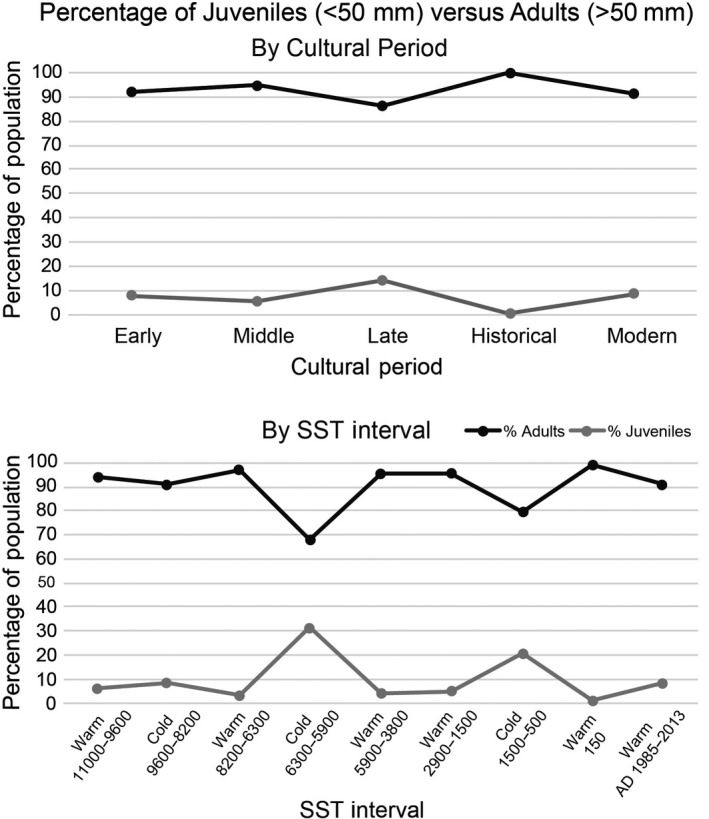
Contribution of adults versus juveniles to the populations of black abalone over time by both temporal period and SST interval. SST: sea surface temperature

Black abalone sizes also varied among SST intervals, with larger sizes observed during warmer SST periods (Figure 4). These differences were significant for all sequential warm–cold comparisons except the earliest two comparisons (Games–Howell post hoc tests: *p* < 0.01; Table 2). Further, abalone size distributions also differed significantly between each successive SST interval, except for the first two (KS tests: *p* < 0.01; Supporting Information Table S3). Specifically, black abalone skewed toward smaller sizes, with peaks in abundance between 50 and 75 mm during each cold‐water interval, but were largely skewed toward larger sizes, with peaks in abundance between 100 and 150 mm, during each warm‐water interval (Figure 5). The relative abundance of adult versus juvenile black abalone was dependent on temporal period and SST interval (contingency table: *p* < 0.01; Table 3). However, this appears more closely influenced by SST than temporal periods, with juveniles making up a higher percentage of the total abalone population during cold intervals than warm ones (Figure 6).

**Table 2:** Results of Games–Howell test analyzing black abalone size (length, mm) by SST interval

Comparison	Mean difference	*SE* of difference	*p*‐Value	Larger period
Warm–Cold (11,000–9,600 to 9,600–8,200 BP)	6.174	2.898	0.459	Warm (11,000–9,600 BP)
Cold–Warm (9,600–8,200 to 8,200–6,300 BP)	7.506	2.729	0.144	Warm (8,200–6,300 BP)
Warm–Cold (8,200–6,300 to 6,300–5,900 BP)	15.729	0.27	<0.01	Warm (8,200–6,300 BP)
Cold–Warm (6,300–5,900 to 5,900–3,800 BP)	33.049	3.890	<0.01	Warm (5,900–3,800 BP)
Warm–Warm (5,900–3,800 to 2,900–1,500 BP)	18.516	1.756	<0.01	Warm (5,900–3,800 BP)
Warm–Cold (2,900–1,500 to 1,500–500 BP)	10.382	1.648	<0.01	Warm (2,900–1,500 BP)
Cold–Warm (1,500–500 to 500–150 BP)	52.241	1.385	<0.01	Warm (500–150 BP)
Warm–Warm (500–150 BP to AD 1985–2013)	24.059	0.905	<0.01	Warm (500–150 BP)

**Table 3:** Numbers and percentages of adult versus juvenile black abalone by temporal period and sea surface temperature (in chronological order)

Period	Adult	Juvenile	Total	% Adult	% Juvenile
Temporal periods					
Early	223	19	242	92.15	7.85
Middle	506	29	535	94.58	5.42
Late	511	83	594	86.03	13.97
Historical	511	2	513	99.61	0.39
Modern	18,793	1,702	20,495	91.70	8.30
Sea surface temperature intervals					
Warm	63	4	67	94.03	5.97
Cold	160	15	175	91.43	8.57
Warm	59	2	61	96.72	3.28
Cold	17	8	25	68.00	32.00
Warm	430	19	449	95.77	4.23
Warm	229	11	240	95.42	4.58
Cold	262	68	330	79.39	20.61
Warm	531	6	537	98.88	1.12
Warm	18,793	1,702	20,495	91.70	8.30

**Figure 7:**
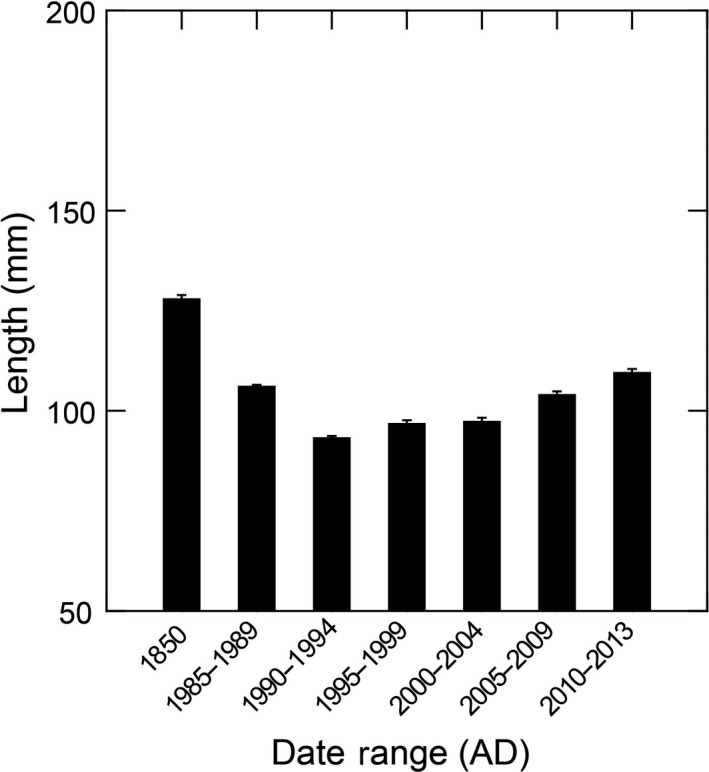
Mean black abalone size (length, mm) through Historical Period and modern 5‐year intervals

During both the Historic and Modern periods, sea otters were locally extirpated and oceanographic conditions were on a warming trend, making comparisons of these datasets important. Archeological research also has documented that historical Chinese abalone fishermen focused on adult black abalone (see Braje, 2016). We, therefore, excluded individuals <50 mm in length from the historical and modern data, and modern data were then separated into 5‐year intervals for detailed time‐series comparison. This identified significant differences in abalone size between the Historic Period and each 5‐year interval of the Modern Period (Games‐Howell: *p* < 0.01; Table 4). Specifically, abalone sizes decreased from the Historic Period through the 1985–1989 modern interval, and then steadily increased since that time (Figure 7).

**Table 4:** Results of Games–Howell test analyzing black abalone size (length, mm) for historical period versus modern period, separated into 5‐year intervals

Comparison (Historical vs. X)	Mean difference	*SE*	*p*‐Value
1985–1989	21.86	0.858	<0.01
1990–1994	34.73	0.903	<0.01
1995–1999	31.12	1.06	<0.01
2000–2004	30.63	1.147	<0.01
2005–2009	23.91	1.064	<0.01
2010–2013	18.42	1.136	<0.01

**Figure 8:**
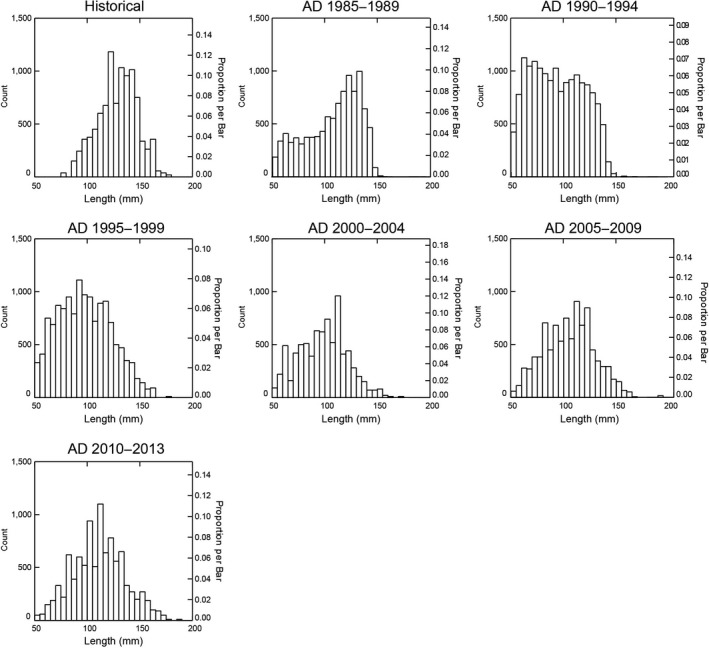
Histogram depicting black abalone population structure for the historic period and modern 5‐year intervals. *Note*: Systat automatically bootstraps histogram data so that the scales are the same for each and can compared visually

Similar to when all ages of abalone were considered, adult abalone size distributions were significantly different between the historical data and each of the six modern intervals (KS tests: *p* < 0.01; Supporting Information Table S4). In particular, sizes observed in the historical data peak between 100 and 150 mm and are skewed toward larger individuals, as are modern data for 1985–1989 and 1990–1994. However, beginning with the 1995–1999 modern interval, the data shift to peak around 100 mm, which continues for the 2000–2004, 2005–2009, and 2010–2013 intervals (Figure 8). For these later intervals, the data are no longer skewed toward larger individuals, but instead trend to a more normal distribution.

## 4 DISCUSSION AND CONCLUSIONS

Similar to previous studies (Braje et al., 2007; Erlandson et al., 2008), we identified little to no obvious temporal trends in mean black abalone size on San Miguel Island during the Early, Middle, and Late Holocene periods. Although we did detect statistically significant differences among these three time periods, we believe these were likely due to our large sample sizes (i.e., 100s), as differences among periods explained very little (15%) of the variation in abalone sizes while natural variability within each period explained 85% of the variation. Indeed, abalone sizes appeared relatively similar across the Holocene, with average shell lengths remaining between ca. 75 and 95 mm with peaks in distributions occurring between 50 and 100 mm. Consequently, increases in human population size, the development of new technologies, the rise of socio‐political complexity, and the resultant increase in human harvesting of abalone throughout the prehistoric period appear to have had little effect on abalone population size distributions. This was surprising as we predicted that a finer‐grained analysis would show that black abalone population distributions would decrease and skew toward smaller individuals over time due to increased harvest of adult abalone associated with growing human populations and fishing pressure. Unlike red abalone (*H. rufescens*), California mussels (*Mytilus californianus*), owl limpets (*Lottia gigantea*), and possibly black turbans (*Chlorostoma funebralis*), there remains no evidence that anthropogenic impacts fundamentally altered black abalone size distributions through the last >10,000 years of Native American fishing (Erlandson et al., 2011, 2008). The effects of intensive human harvesting of black abalone may have occurred before this time, but we currently have little or no black abalone data from sites older than 10,000 years. However, during the Historic Period, when predation on abalone by humans and otters was reduced, average sizes increased dramatically to more than 125 mm and the population distributions peaked between 125 and 150 mm. Then, during the Modern Period when commercial harvesting began and disease impacted the abalone populations, average sizes decreased to 97 mm and distributions peaked between 100 and 150 mm. Together, this suggests that predation and disease in the periods following the Holocene did impact abalone sizes, especially on larger individuals.

When data were grouped by SST interval, additional patterns emerged. Except for the earliest warm and cold intervals, there were significant differences in black abalone size distributions between warm versus cold SST intervals. Populations cluster around smaller sizes and skew toward smaller individuals (50–75 mm) during cold‐water intervals than during warm internals (>100 mm). Consistent with this, the contribution of juveniles versus adults is larger for cold‐water intervals than warm intervals, but the removal of juveniles from the dataset did not alter our results. This finding is surprising as modern abalone show decreased growth rates during warm‐water periods, such as ENSO events, due to decreased supplies of giant kelp (Day & Fleming, 1992; Edwards & Estes, 2006). As Day and Fleming (1992) noted, however, studies of SST on abalone growth are often impaired by short time periods and a number of factors that may influence growth such as algae and kelp availability, temperature, spawning periods, and densities of conspecifics, competitors, and predators may have played significant roles over longer time intervals.

Over longer time spans, the effects of SST on abalone growth and size may be more equivocal. Increases in abalone sizes during short cold‐water intervals may result from less interspecies competition for space and food, as food consumption in some abalone species is positively related to water temperature (Britz, Hecht, & Mangold, 1997). Macroalgae are generally more productive during cold SST periods when nutrient levels are high, and abalone grow faster and are more abundant due to increased algal drift (Tissot, 1990). This can lead to their aggregation in larger groups, however, where they exhibit stacking that reduces mobility and increases competition for food (Duros, 1987). As a result, such abalone can exhibit lower growth rates and smaller overall sizes (e.g., Lloyd & Bates, 2008). In contrast, during long periods of warmer SSTs over centennial to millennial scales, some competitors and predators may be better equipped to cope with elevated temperatures than other intertidal species (e.g., Fly, Monaco, Pincebourde, & Tullis, 2012; Sanford, 2002;), though they also may be more susceptible to disease (Ben‐Horin, Lenihan, & Lafferty, 2013). It is also possible that other intertidal species may be more productive during prolonged cold‐water periods and present a more enticing prey than abalone, leading to decreased predatory pressure and increased size. In addition, during warm SST periods there may have been a shift in prey species due to increased availability. Study of abalone growth rates and survivorship under typical short‐term biological experiments and observations is unlikely to identify such patterning. Our data indicate very different trends when observing changes in black abalone size over longer timescales, underscoring the importance of understanding the aggregation of black abalone, in addition to individual size, for interpreting population health.

Although there seems to be a shift in modern black abalone size structure toward distributions similar to those in ancient times, modern populations still skew in comparison to prehistoric patterns, which appear to have been relatively stable for ~10,000 years until population collapse in the late twentieth century. Our results suggest that baselines constructed from historical commercial catch records alone are not sufficient for the long‐term management of abalone. Abalone populations increased greatly during the historical period, reaching sizes and densities not seen during the previous, and seemingly more stable, deep history of Native American black abalone fishing. These data provide a measure against which modern population distributions can be evaluated and offer an opportunity to build management protocols based on human‐abalone ecodynamics over the *longue durée*.

## CONFLICT OF INTEREST

None declared.

## AUTHOR CONTRIBUTIONS

HH and TB conceptualized and drafted the manuscript. ME, JE, and SW rewrote and drafted sections, edited, and shared essential data.

## Data Availability

Our data is available open source via Dryad at https://doi.org/10.5061/dryad.8m3t2p2.
