# Supplementary figures and images for: Black abalone (Haliotis cracherodii) population structure shifts through deep time: Management implications for southern California's northern Channel Islands
Source: Ecol Evol. 2019 Apr 2;9(8):4720–32. doi: 10.1002/ece3.5075 (PMC6476767; doi:10.1002/ece3.5075)

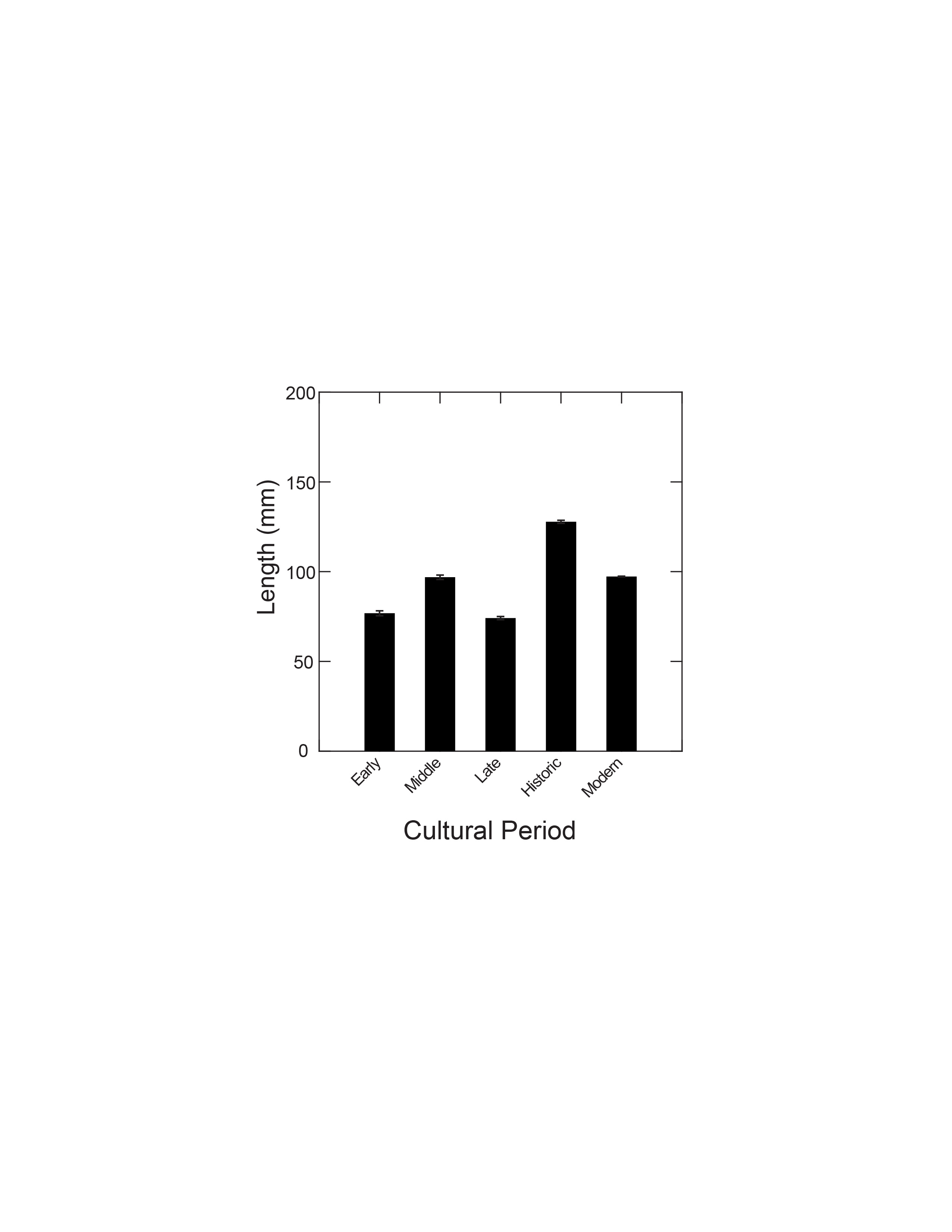

Supplement: Supplementary file 1 [file ECE3-9-4720-s001.png]
