# Supplementary material for: Black abalone (Haliotis cracherodii) population structure shifts through deep time: Management implications for southern California's northern Channel Islands
Source: Ecol Evol. 2019 Apr 2;9(8):4720–32. doi: 10.1002/ece3.5075 (PMC6476767; doi:10.1002/ece3.5075)
Supplement: Supplementary file 2 [file ECE3-9-4720-s002.docx]

Supplementary Tables:

Supplementary Table 1. Results of Games-Howell test analyzing black abalone size by temporal period.

| Comparison | Mean Difference | Std. Error of Difference | P-Value | Larger Period |
| --- | --- | --- | --- | --- |
| Early-Middle | 19.952 | 1.683 | <0.01 | Middle |
| Middle-Late | 20.769 | 1.409 | <0.01 | Middle |
| Late-Historical | 48.948 | 1.669 | <0.01 | Historical |
| Historical-Modern | 25.792 | 0.841 | <0.01 | Historical |

Supplementary Table 2. Results of KS tests analyzing black abalone size by temporal period.

| Comparison | KS Z | P-Value |
| --- | --- | --- |
| Early-Middle | 4.736 | <0.01 |
| Middle-Late | 6.018 | <0.01 |
| Late-Historical | 13.242 | <0.01 |
| Historical-Modern | 9.614 | <0.01 |

Supplementary Table 3. Results of KS test analyzing black abalone size (length, mm) by SST interval.

| Comparison | KS Z | P-Value |
| --- | --- | --- |
| Warm- Cold (11000-96000 to 9600-8200 BP) | 1.117 | 0.165 |
| Cold –Warm (9600-8200 to 8200-6300 BP) | 1.705 | <0.01 |
| Warm-Cold (8200-6300 to 6300-5900 BP) | 2.311 | <0.01 |
| Cold-Warm (6300-5900 to 5900-3800 BP) | 3.134 | <0.01 |
| Warm-Warm (5900-3800 to 2900-1500 BP) | 4.081 | <0.01 |
| Warm-Cold (2900-1500 to 1500-500 BP) | 3.818 | <0.01 |
| Cold-Warm (1500-500 to 500-150 BP) | 11.713 | <0.01 |
| Warm-Warm (500-150 BP to AD 1985-2013) | 9.083 | <0.01 |

Supplementary Table 4. Results of KS test analyzing black abalone size (length, mm) for the historical period versus modern five-year intervals.

| Comparison (Historical vs. X) | Mann-Whitney U | P-Value |
| --- | --- | --- |
| 1985-1989 | 1344018.5 | <0.01 |
| 1990-1994 | 308044.50 | <0.01 |
| 1995-1999 | 120680.50 | <0.01 |
| 2000-2004 | 61726.50 | <0.01 |
| 2005-2009 | 141293.50 | <0.01 |
| 2010-2013 | 138107.50 | <0.01 |
